# Manipulation of working memory contents selectively impairs metacognitive sensitivity in a concurrent visual discrimination task

**DOI:** 10.1093/nc/niv002

**Published:** 2015-07-03

**Authors:** Brian Maniscalco, Hakwan Lau

**Affiliations:** ^1^ Department of Psychology, Columbia University;; ^2^ National Institute of Neurological Disorders and Stroke, National Institutes of Health;; ^3^ Department of Psychology, UCLA

**Keywords:** metacognition, working memory, psychophysics, signal detection theory

## Abstract

What are the cognitive mechanisms underlying perceptual metacognition? Prior research indicates that prefrontal cortex (PFC) contributes to metacognitive performance, suggesting that metacognitive judgments are supported by high-level cognitive operations. We explored this hypothesis by investigating metacognitive performance for a visual discrimination task in the context of a concurrent working memory (WM) task. We found that, overall, high WM load caused a nonspecific decrease in visual discrimination performance as well as metacognitive performance. However, active manipulation of WM contents caused a selective decrease in metacognitive performance without impairing visual discrimination performance. These behavioral findings are consistent with previous neuroscience findings that high-level PFC is engaged by and necessary for (i) visual metacognition, and (ii) active manipulation of WM contents, but not mere maintenance. The selective interference of WM manipulation on metacognition suggests that these seemingly disparate cognitive functions in fact recruit common cognitive mechanisms. The common cognitive underpinning of these tasks may consist in (i) higher-order re-representation of lower-level sensory information, and/or (ii) application of decision rules in order to transform representations in PFC into definite cognitive/motor responses.

## Introduction

Perceptual metacognition refers to the capacity of human and animal observers to introspectively differentiate perceptual judgments that are likely to be correct from those that are less likely to be correct. What are the cognitive and neural mechanisms underlying perceptual metacognition? Several lines of evidence link parts of the prefrontal cortex (PFC),including dorsolateral prefrontal cortex (dlPFC), rostrolateral prefrontal cortex (rlPFC) and anterior prefrontal cortex (aPFC), to metacognition in the visual domain, as well as in the domain of memory: activations in dlPFC and rlPFC have been found to inversely correlate with reports of confidence in visual and memory tasks (Henson *et al.*, 2000; Fleck *et al.*, 2006; Fleming *et al.*, 2012), and rlPFC activations have been found to correlate with metacognitive sensitivity in visual (Fleming *et al.*, 2012) and memory (Yokoyama *et al.*, 2010) tasks. Similarly, single unit recording activity in macaque aPFC has been shown to increase the following correct decisions in a cuing task, even before task feedback is provided (Tsujimoto *et al.*, 2010). In humans, individual differences in aPFC gray matter volume positively correlate with visual metacognitive sensitivity (Fleming *et al.*, 2010; McCurdy *et al.*, 2013). Direct evidence for the causal role of PFC in visual metacognition is provided by transcranial magnetic stimulation and lesion studies: disruption of bilateral dlPFC via transcranial magnetic stimulation selectively impairs visual metacognition while leaving visual discrimination performance intact (Rounis *et al.*, 2010), and patients with aPFC lesions exhibit selective deficits in visual metacognition, as compared with temporal lobe lesioned patients and healthy controls (Fleming *et al.*, 2014).

dlPFC is also involved in working memory (WM) performance. Multiple lines of evidence implicate dlPFC particularly in the active processing of WM contents, rather than the mere storage of WM contents, which is typically attributed to more posterior brain regions, e.g. parietal and occipital cortex (Petrides, 2000; Miller and Cohen, 2001; Curtis and D'Esposito, 2003). For instance, dlPFC activations during delay periods in WM tasks increase when the task requires WM contents to be manipulated (D'Esposito *et al.*, 1999), and other studies have found that dlPFC does not preferentially activate during delay periods, but rather its activation profile reflects the specific process of response selection performed on the basis of WM contents (Rowe *et al.*, 2000; Rowe and Passingham, 2001;Rowe *et al.*, 2002). Basic short-term memory performance can be spared in patients with bilateral prefrontal damage (Petrides, 1989; Owen *et al.*, 1996), but dlPFC lesions impair performance on tasks that require active monitoring and manipulation of WM contents in humans (Petrides and Milner, 1982) and macaques (Petrides, 1995). dlPFC also becomes more activated in WM tasks in which a cognitive strategy allows WM contents to be “chunked” into higher-level units, even though such chunking strategies effectively reduce the number of “items” in WM (Bor *et al.*, 2003). This finding again suggests that dlPFC is more closely linked to strategic monitoring and manipulation of WM contents than it is to the overall difficulty of the memory task or to the number of items that need to be stored in WM.

Given that PFC is recruited in both visual metacognition and executive processing of WM contents, it is possible that common underlying mechanisms are at play in both kinds of cognitive functions. If so, we might expect that metacognitive performance would be selectively impaired by concurrently manipulating WM contents, especially in light of general processing capacity limits and bottlenecks in PFC (Marois and Ivanoff, 2005). Here we test this hypothesis in a dual-task paradigm. While holding a letter string in memory and alphabetizing it, subjects performed a simple two-alternative forced choice visual task and provided confidence ratings. After the visual task, a probe assessed memory for the alphabetized string. We analyzed metacognitive performance under low and high WM load. Within the high WM load condition, we further distinguished between trials that placed low and high manipulation demand (i.e. strings requiring little or extensive alphabetization). To anticipate, we found that metacognitive performance was selectively impaired under high WM load with high manipulation demand, suggesting that a common mechanism contributes to metacognitive evaluation of perceptual decision making and active manipulation of WM contents.

## Materials and methods

### Experiment 1

#### Participants

Twenty-three Columbia University students participated in the experiment. Participants gave informed consent and were paid $10 for ∼1 h of participation. The research was approved by the Columbia University’s Committee for the Protection of Human Subjects.

One participant was omitted from data analysis, due to producing outlying data in the perceptual metacognitive task under high WM load (see “Exclusion of outliers” in Materials and methods and Fig. 4).

#### Experimental procedure

Subjects were seated in a dimmed room 60 cm away from a computer monitor. Stimuli were generated using Psychophysics Toolbox (Brainard, 1997; Pelli, 1997) in MATLAB (MathWorks, Natick, MA) and were shown on an iMac monitor (LCD, 24 inches monitor size, 1920 x 1200 pixel resolution, 60 Hz refresh rate).

**Figure 1:**
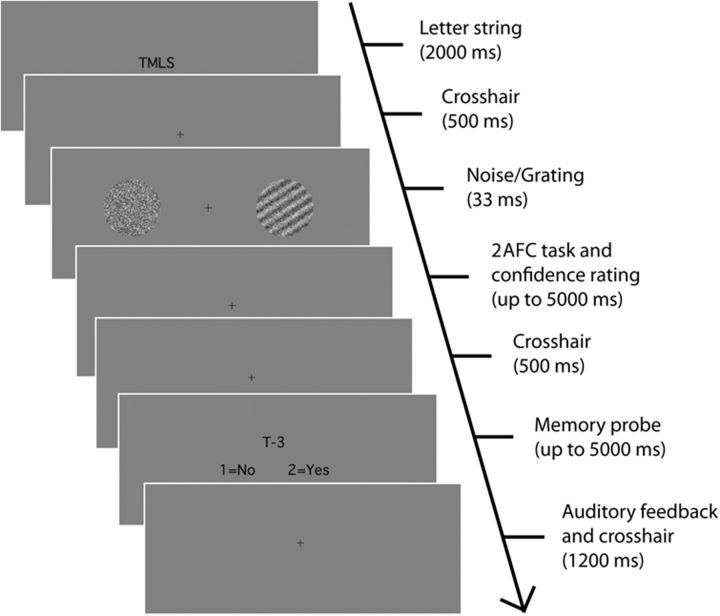
Experimental design Subjects performed a WM task concurrently with a perceptual decision making task. At the start of the trial, a letter string was presented. Subjects were informed to hold the string in memory and sort it into alphabetical order. Strings could be either one letter long (Low Load) or four letters long (High Load). Due to randomization of the four letter strings, these could be either easy to alphabetize (High Load – Easy) or difficult (High Load – Hard). Subsequently, subjects performed a spatial two-alternative forced-choice (2AFC) task. Two noisy stimuli appeared to the left and right of fixation, and one of these contained a sinusoidal grating. Subjects indicated which side the grating appeared on and rated decision confidence on a scale of 1–4. Finally, subjects performed the WM task. A memory probe consisting of a letter-number pair inquired as to whether the probe-letter was located at probe-number position of the alphabetized string. Experiments 1 and 2 used this same basic design, with slight modifications between them (see Materials and methods).

On every trial, a WM task was performed concurrently with a visual discrimination task (Fig. 1). At the start of the trial, an uppercase letter string in black font was displayed on a gray background for 2000 ms. The letter string could consist of either one letter (Low Load condition) or four letters (High Load condition). The across-trial sequence of one- and four-letter string presentations was randomized, such that each string size occurred with equal frequency. Letters in the four-letter strings were presented in random alphabetical order. Letters were chosen randomly from the following set of letters: (F, G, H, J, K, L, M, N, P, Q, R, S, T). Vowels and letters early and late in the alphabet were omitted to increase memorization and alphabetization difficulty. Subjects were instructed to hold the letter string presented at the start of the trial in memory and to alphabetize it, since memory for the alphabetized string would be probed at the end of the trial. This task design closely follows designs used previously to compare the effects of manipulating, versus passively maintaining, WM contents (D'Esposito *et al.*, 1999; Han and Kim, 2004).

After the letter string was presented, a crosshair (0.35° wide) was presented centrally for 500 ms, and then the stimuli for the visual discrimination task were presented. Two stimuli were presented simultaneously for 33 ms, one 4° to the left of fixation and one 4° to the right. Each stimulus was a circle (3° diameter) consisting of randomly generated visual noise. The target stimulus contained a randomly oriented sinusoidal grating (two cycles per degree) embedded in the visual noise. After stimulus presentation, subjects provided a forced-choice judgment of whether the left or the right stimulus contained a grating. The grating location was determined randomly on each trial, and gratings appeared equally often on the left and right. Following stimulus classification, subjects rated their confidence in the accuracy of their response on a scale of 1 through 4. Subjects were encouraged to use the entire confidence scale. If the confidence rating was not registered within 5 s of stimulus offset, the trial proceeded as if a confidence rating had been entered. Such trials were omitted from all analyses. There was a 500-ms interval between the entry of confidence rating and the presentation of the memory probe.

The memory probe consisted of a letter and a number, e.g. “T-3.” Subjects judged whether it was true that the letter of the memorized and alphabetized string picked out by the probe number matched the probe letter. For instance, suppose that the initial letter string was “TMLS,” and the memory probe was “T-3.” The “T-3” probe would pose the question, “is it true that the 3^rd^ letter in the alphabetized letter string is a T?” Subjects indicated either “yes” or “no” in response to the probe. In this example, the correct answer is “no,” since the alphabetized string is “LMST”, and the third letter of this string is S, not T. Probe letters were always selected randomly from one of the letters contained in the original letter string. As a consequence of this policy, for one-letter strings, the correct answer was always “yes.” For four-letter strings, the probe letter was chosen randomly. For half of all trials, the probe number corresponded to the true index of the probe letter in the alphabetized string. For the remaining half of all trials, the probe number was chosen randomly from one of the three remaining indices. Thus, for four-letter strings, the correct answer was “yes” for half of all the trials. Grating location, letter string size and correct answer for four-letter strings (“yes” or “no”) were counterbalanced.

If no memory response was entered within 5 s of probe onset, the trial proceeded as if a response had been entered. Such trials were omitted from all analyses. After entry of the memory response, a crosshair was presented centrally for 1200 ms. At the beginning of this interval, a 200-ms tone indicated accuracy for the WM task—a brief high-pitched tone indicated a correct memory response, and a brief low-pitched tone indicated an incorrect response. After this 1200 ms interval passed, the letter string for the next trial was presented. Although subjects were encouraged to do their best on both the visual and memory task, they were not explicitly instructed to prioritize one task over the other. However, it is possible that the task structure implicitly encouraged subjects to prioritize the memory task, since performance feedback was provided only for the memory task.

At the start of each experimental session, subjects completed two practice blocks (20 trials each) and one calibration block (120 trials). In the calibration block, performance on the spatial two-alternative forced choice grating localization task was adjusted continuously between trials on the basis of the subject’s task performance using the QUEST threshold estimation procedure (Watson and Pelli, 1983). In order to shorten trial length, no letter strings or memory probes were presented during the calibration block; each trial consisted only of presentation of the visual stimuli, followed by the subject’s key presses indicating the grating location and decision confidence. Target stimuli were defined as the sum of a grating with Michelson contrast C_grating_ and a patch of visual noise with Michelson contrast C_noise_. The total contrast of the target stimulus, C_target_ = C_grating_ + C_noise_, was set to 0.9. The nontarget stimulus containing only noise was also set to a Michelson contrast of 0.9. The QUEST procedure was used to estimate the ratio of the grating contrast to the noise contrast, R_g/n_ = C_grating_/C_noise_, which yielded 72% correct performance in the 2IFC task. Three independent threshold estimates of R_g/n_ were acquired, with 40 randomly ordered trials contributing to each, and the median estimate of these was used to create stimuli for the main experiment.

In the main experiment, subjects completed eight blocks of 50 trials each, for a total of 400 trials. After each block, subjects were provided with a self-terminated rest period lasting up to 1 min.

### Experiment 2

#### Participants

Thirty Columbia University students participated in the experiment. Participants gave informed consent and were paid $10 for approximately 1 h of participation. The research was approved by the Columbia University’s Committee for the Protection of Human Subjects.

One participant was omitted from data analysis, due to producing outlying data in the perceptual metacognitive task under high WM load (see “Exclusion of outliers” in Materials and methods and Fig. 4).

#### Experimental procedure

The experimental procedure was identical to that of Experiment 1, with three exceptions. These modifications were made to address several inadequacies in Experiment 1.

First, in the WM task, the probe letter was now allowed to differ from the original letter string for one-letter strings. For one-letter strings, the probe letter matched the original letter for half of all trials, and thus the correct answer for the memory task was “yes” on half of all trials. This change ensured that the Low Load WM task could not be performed trivially by always answering “yes,” as was the case in Experiment 1, and thus that active engagement of memory was required to achieve high performance. As in Experiment 1, however, the probe letter for four-letter strings was always randomly selected from one of the letters contained in the initially presented string.

Second, in the visual discrimination task, two levels of grating contrast, instead of one single level, were used. For the higher level of grating contrast, the ratio R_g/n_ of grating contrast to noise contrast yielding 72% correct performance in the visual 2AFC task was determined in the calibration block, as in Experiment 1. For the lower level of grating contrast, R_g/n_ was set to half of the value used for higher grating contrast stimuli. As with the high grating contrast stimuli, the low grating contrast stimuli were defined by adding the low-contrast grating to a white noise pattern, such that the contrast of the grating+noise stimulus as a whole was set to 0.9. Contrast level was counterbalanced with grating location, letter string size and correct answer for the memory task (“yes” or “no”). This change was intended to shed light on the empirical relationship between *d’* and meta-*d’* by allowing us to measure these variables at two levels of task performance for each subject.

Third, the presentation of one- and four-letter strings was now blocked, rather than randomly interleaved across trials. For 14 subjects, the first 4 blocks (200 trials) of the main experiment contained only one-letter strings, and the last 4 blocks (200 trials) contained only four-letter strings. For the remaining 16 subjects, the order was reversed. Assignment of subjects to the low-load-first and high-load-first conditions was randomized. This change was intended to strengthen the effect of WM load on visual task performance; presumably, any potential effects of high WM load on visual task performance might be potentiated by consistently applying High Load over a long period of time, rather than randomly interleaving high and Low Load trials.

#### Data analysis for the perceptual task

We measured perceptual and metacognitive performance in the visual task using signal detection theory (SDT) analysis (Green and Swets, 1966; Macmillan and Creelman, 2005; Maniscalco and Lau, 2014). We defined hit rate (HR) as the probability that the subject reported that the grating was on the right, given that the grating was on the right, and false alarm rate (FAR) as the probability that the subject reported that the grating was on the right, given that the grating was on the left. We calculated *d’* = z(HR) – z(FAR) and used *d’* to quantify sensitivity in the visual discrimination task.

We similarly quantified metacognitive sensitivity, i.e. the efficacy with which confidence ratings discriminate between a subject’s own correct and incorrect responses, with meta-*d’* (Maniscalco and Lau, 2012, 2014). Specifically, for each WM condition of each subject’s data, we found the value of meta-*d’* that jointly maximized the likelihood of the response-specific type 2 relative operating characteristic (ROC) curves, where response-specific type 2 ROC curves are derived from “type 2” probabilities of the general form P(confidence = c | stimulus = s and response = r) for all possible values of confidence (1 – 4), stimulus (“grating on left” or “grating on right”) and response (“grating was on the left” or “grating was on the right”) occurring in this experiment. Maximization of likelihood was achieved using the Optimization Toolbox in MATLAB (MathWorks, Natick, MA). Essentially, estimating meta-*d’* in this analysis amounts to fitting the SDT model to all such type 2 probabilities for each experimental condition of each subject’s data. Please see Maniscalco and Lau(2012, 2014) for a more in-depth treatment of the methodology for estimating meta-*d’*.

According to SDT, perceptual sensitivity and metacognitive sensitivity are directly correlated; as an observer becomes better at performing a perceptual tasks, it theoretically follows that metacognitive sensitivity also improves (Galvin *et al.*, 2003; Maniscalco and Lau, 2012). Meta-*d’* is defined such that, if an observer with perceptual sensitivity *d’* exhibits metacognitive performance exactly in line with the SDT prediction, then meta-*d’* = *d’*. However, if the observer underperforms SDT expectation, then meta-*d’* < *d’*.

As discussed in Maniscalco and Lau (2012), these observations suggest a useful conceptual distinction between ‘absolute’ and ‘relative’ metacognitive sensitivity. Absolute metacognitive sensitivity concerns how well confidence ratings discriminate correct from incorrect responses overall. Relative metacognitive sensitivity concerns how well confidence ratings discriminate correct from incorrect responses, ‘relative’ to how informative we might expect those confidence ratings to be in light of the observer’s perceptual performance. Whereas absolute metacognitive sensitivity can be measured straightforwardly with meta-*d’*, relative metacognitive sensitivity can be measured by means of a numerical comparison between meta-*d’* and *d’*. Relative metacognitive sensitivity is a useful construct in that it allows us to take the theoretical relationship between perceptual and metacognitive performance into account when evaluating metacognitive performance, which in turn facilitates discovery of “genuinely” metacognitive effects, as opposed to differences in absolute metacognitive sensitivity that can potentially be attributed to differences in the underlying perceptual task performance.

In Experiment 2, the low grating contrast condition led to unexpectedly low levels of performance in the perceptual task. Average *d’* for the low grating contrast stimuli was 0.25, which for an unbiased observer corresponds to a rate of 55% correct responding. One sample *t*-tests revealed that both *d’* and meta-*d’* were significantly >0 (i.e. the chance level of responding) in the low-contrast condition (*p*s < 0.05). However, at these near-chance levels of performance, data are noisy and subject to floor effects. For these reasons, we focus on analyzing only the high-contrast condition of Experiment 2.

Nonetheless, the data from the low contrast condition are useful to the extent that they demonstrate that meta-*d’* scales directly with *d’* and can be significantly better than chance even when *d’* is itself close to chance performance. In turn, this suggests that the function relating *d’* and meta-*d’* has a y-intercept approximately equal to zero. On the assumption that the function relating *d’* and meta-*d’* is linear (with a zero y-intercept), the slope of this line would then indicate the quality of metacognitive performance relative to perceptual performance. But the slope of a line with zero y-intercept is just the ratio of y to x. Therefore, computing the ratio meta-*d’* / *d’* is akin to measuring the slope of the line relating meta-*d’* and *d’,* and thus provides a means of measuring the quality of metacognitive performance, relative to perceptual performance. We therefore compute M_ratio_ = meta-*d’*/*d’* to measure relative metacognitive sensitivity.

#### Exclusion of outliers

Inspection of the data entering into the analysis of M_ratio_ as a function of WM condition revealed that Experiment 1 and Experiment 2 each had a single subject with outlying data (Fig. 4). To quantify this effect, we computed M_D, L_ = M_Low Load_ – M_High Load – Hard_ and M_D, H_ = M_High Load – Easy_ – M_High Load – Hard_ for each subject and converted these measures into z-scores separately for Experiments 1 and 2. We then computed the average of the z-scores for M_D, L_ and M_D, H_ and used this as an index for quantifying the degree to which each subject produced outlying data for M_ratio_ as a function of WM condition. The subjects marked as outliers in Fig. 4 had combined z-scores of −3.59 and 3.59, respectively. Across both experiments, no other subject had a combined z-score with an absolute value >1.7. Thus, we excluded the two subjects with extreme combined z-scores from all analyses reported here.

## Results

Due to the similarities in experimental design and empirical outcomes in Experiments 1 and 2, we will present the results from these experiments concurrently. The primary analysis of interest is to assess performance in the perceptual task as a function of difficulty of the WM task. We therefore distinguish between Low Load (one-letter memory string) and High Load (four-letter memory string) conditions.

We further categorize the four-letter strings by the degree to which these randomly created strings were initially presented in alphabetical order. Since subjects were required not only to hold the strings in memory but also to alphabetize them, the degree to which the strings were initially well-alphabetized or scrambled could further modulate the resources or processes required to perform the memory task. We classified string alphabetization by counting how many of the three consecutive letter pairs in each four-letter string were in alphabetical order. For instance, in the string ADBC, two consecutive letter pairs are in alphabetical order (AD and BC) but one is not (DB). Strings with two or three letter pairs in alphabetical order were considered to be well alphabetized, and strings with zero or one letter pair in alphabetical order were considered to be poorly alphabetized. We use the term High Load—easy to refer to trials with well-alphabetized four letter strings, and the term High Load—hard to refer to trials with poorly alphabetized four letter strings, where “Easy” and “Hard” denote the difficulty of the alphabetization task.

### WM *performance*

**Figure 2:**
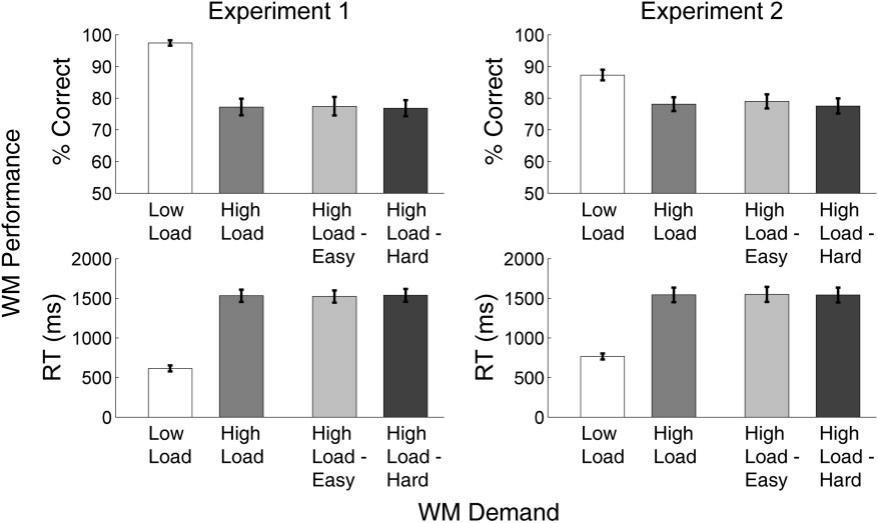
WM performance As expected, the memory task was significantly more difficult under High Load than under Low Load, exhibiting significantly lower rates of correct responding and longer reactions times. However, within the High Load condition, the distinction between easy and difficult alphabetization did not manifest as an observable change in WM task performance. Error bars represent 1 SEM.

Overall, the WM load manipulation was successful in presenting a challenging WM task (Fig. 2), as revealed by separate 2 (WM Load: High, Low) x 2 (Experiment: 1, 2) mixed-measures analysis of variances (ANOVAs) on accuracy and reaction time in the WM task. Compared to the Low Load condition, High Load decreased accuracy [main effect of WM load, F(1, 49) = 73.17, *P* < 0.001; Experiment 1: Low Load mean = 97.4% correct, SEM = 1.6%; High Load mean = 77.8% correct, SEM = 2.6%; Experiment 2: Low Load mean = 88.4% correct, SEM = 1.4%; High Load mean = 77.7% correct, SEM. = 2.1%] and increased median reaction time [WM load, F(1, 49) = 238.24, *P* < 0.001; Expt 1: Low Load mean = 609 ms, SEM = 40 ms; High Load mean = 1537 ms, SEM = 96 ms; Expt 2: Low Load mean = 761 ms, SEM = 32 ms; High Load mean = 1519 ms, SEM = 78 ms]. However, alphabetization difficulty did not affect accuracy (*P* = 0.4) or median reaction time (*P* > 0.9) in the memory task.

The main effect of WM load on task performance was modulated by a significant WM Load x Experiment interaction [F(1, 49) = 9.53, *P* = 0.003]. The source of this interaction was that memory performance under Low Load was significantly better in Experiment 1 than in Experiment 2 [independent samples *t*-test on % correct, *t*(49) = 4.72, Bonferroni corrected *P* < 0.001]. (Median reaction time on the memory task was also faster in Experiment 1, although the WM Load x Experiment interaction for median RT did not achieve significance, *P* = 0.19.) This difference was due to the fact that the Low Load task was trivial in Experiment 1, as the probe letter was always the same as the one-letter string, whereas in Experiment 2, the probe only matched the one-letter string on half of all trials and thus posed a simple but nontrivial memory demand (see Materials and methods). However, the structure of the memory task under High Load was identical for the two experiments, and here memory performance did not differ for either accuracy or reaction time (*p*s > 0.8).

### Perceptual task performance as a function of WM load

**Figure 3:**
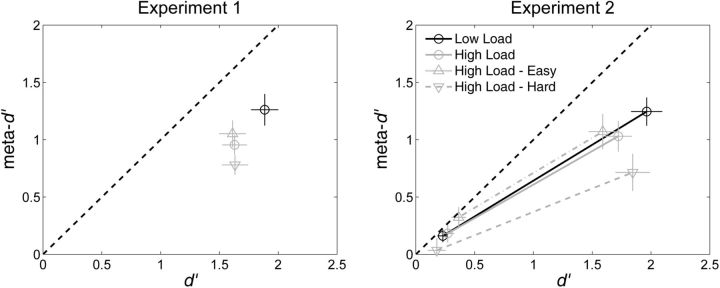
Perceptual and metacognitive performance as a function of WM load We measured perceptual sensitivity with the SDT measure *d’* (Green and Swets, 1966) and metacognitive sensitivity with the SDT measure meta-*d’* (Maniscalco and Lau, 2012, 2014). If subjects performed according to SDT expectations, data should fall along the dashed line of unity, meta-*d’* = *d’*. As expected, subjects’ metacognitive sensitivity underperformed SDT expectation. Overall, under High Load, *d’* and meta-*d’* were equally impaired. Crucially, well-scrambled WM strings were associated with an impaired *ratio* of meta-*d’* to *d’*, suggesting that the process of manipulating the contents of WM had a selective deficit on relative metacognitive sensitivity. On these plots, this result manifests as the data for the High Load – Hard condition occupying a lower region on the y-axis of the meta-*d’* vs *d’* plot than the other data points in spite of having a similar *x*-axis value. In the plot for Experiment 2, data from the low stimulus contrast condition is displayed in the lower left region of the plot; these data were not included in subsequent analyses due to excessively low values for *d’* (see Materials and methods). Error bars represent 1 SEM.

Figure 3 displays *d’* and meta-*d’* as a function of WM load and alphabetization difficulty. We analyzed this data with separate 2 (WM Load: High, Low) x 2 (Experiment: 1, 2) mixed design ANOVAs for *d’* and meta-*d’*. In both experiments, WM Load impaired perceptual sensitivity [*d’*; WM Load, F(1, 49) = 14.11, *P* < 0.001; WM Load x Experiment, *P* > 0.9; Expt 1: Low Load mean = 1.88, SEM = 0.14; High Load mean = 1.63, SEM = 0.12; Expt 2: Low Load mean = 1.96, SEM = 0.12; High Load mean = 1.72, SEM = 0.11] and metacognitive sensitivity [meta-*d’*; WM Load, F(1, 49) = 9.25, *P* = 0.004; WM Load x Experiment, *P* = 0.6; Expt 1: Low Load mean = 1.26, SEM = 0.14; High Load mean = 0.95, SEM = 0.13; Expt 2: Low Load mean = 1.25, SEM = 0.12; High Load mean = 1.03, SEM = 0.11].

**Figure 4:**
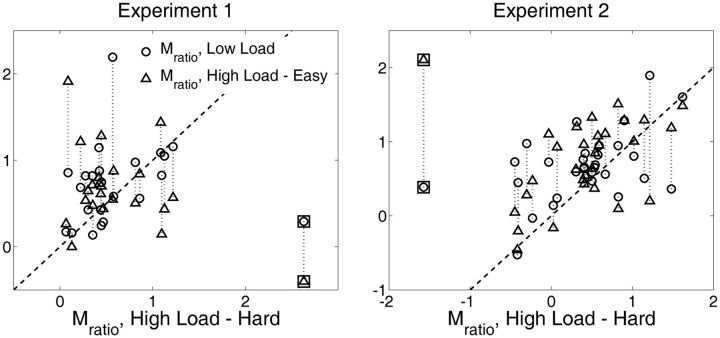
Scatterplots of M_ratio_ under the different WM conditions M_ratio_, as the ratio of meta-*d’* to *d’*, measures how well the subject performed metacognitively (meta-*d’*) in relation to perceptual performance (*d’*). For subjects behaving according to SDT expectation, M_ratio_ = 1, whereas M_ratio_ < 1 indicates metacognitive performance that is suboptimal relative to SDT expectation. The scatterplots display the relationship between M_ratio_ under the Low Load and High Load – Easy conditions (plotted on the *y*-axis) to M_ratio_ under the High Load – Hard condition (plotted on the *x*-axis). Dashed vertical lines connect the two data points on each plot generated by the same subject. Most points fall above the line of unity, suggesting that M_ratio_ is impaired in the High Load – Hard condition compared to the Low Load and High Load – Easy conditions. Data with icons contained inside squares were considered to be outliers due to having very large z-scores (see “Exclusion of outliers” in Materials and methods), and data from these two subjects was omitted from all analyses. In Experiment 1, after excluding outliers, M_ratio_ values were significantly correlated under the High Load – Hard and Low Load conditions (*r* = 0.42, *P* = 0.05) but not under the High Load – Hard and High Load – Easy conditions (*r* = −0.1, *P* = 0.7). In Experiment 2, M_ratio_ under the High Load – Hard conditions significantly correlated with M_ratio_ under Low Load (*r* = 0.5, *P* = 0.006) and High Load – Easy (*r* = 0.61, *P* < 0.001).

However, the reduction in meta-*d’* due to WM load is qualified by the fact that WM load also reduced *d’*. Since *d’* and meta-*d’* theoretically correlate (Galvin *et al.*, 2003; Maniscalco and Lau, 2012), the reduction in meta-*d’* under high WM load might be attributable merely to the reduction in *d’*, rather than to a direct, independent effect on metacognitive performance per se. If WM load impaired metacognitive performance over and above its impairment of perceptual performance, we might expect that WM load would decrease the ratio meta-*d’*/*d’*, which we shall hereafter refer to as M_ratio_. Although M_ratio_ was numerically lower under High Load (Expt 1: Low Load mean = 0.74, SEM = 0.10; High Load mean = 0.61, SEM = 0.08; Expt 2: Low Load mean = 0.68, SEM = 0.09; High Load mean = 0.63, SEM = 0.07), these differences were not statistically significant in the WM Load x Experiment ANOVA (WM Load, *P* = 0.16; WM load x Experiment, *P* > 0.5). Thus, we did not find compelling statistical evidence that overall WM load reduces relative metacognitive sensitivity, as measured by M_ratio_.

### Perceptual task performance as a function of WM load and alphabetization difficulty

In order to take into account the effect of alphabetization difficulty, we calculated M_ratio_ separately for the High Load – Easy and High Load – Hard conditions. Scatterplots relating M_ratio_ for these conditions as well as M_ratio_ under Low Load are displayed in Fig. 4. One subject in each of the Experiments 1 and 2 produced outlying data on these plots, and were therefore excluded from all analyses. Inspection of the remaining data suggest that M_ratio_ was lower under the High Load – Hard condition than in the High Load – Easy and Low Load conditions.

**Figure 5:**
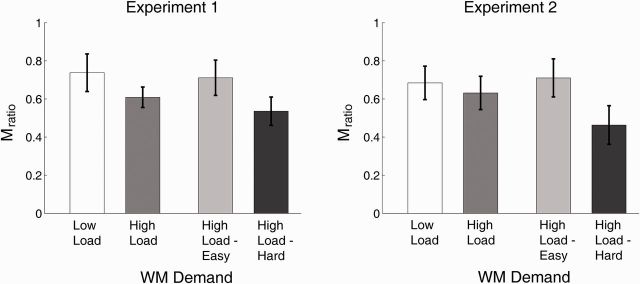
Average values of M_ratio_ across WM load conditions In both experiments, although M_ratio_ was numerically lower under High Load than under Low Load, the difference was not statistically significant. However, M_ratio_ under the High Load – Hard condition was significantly lower than it was under Low Load and High Load – Easy conditions, suggesting that active manipulation of WM contents selectively disrupts visual metacognitive sensitivity. In contrast, M_ratio_ did not differ between the Low Load and High Load – Easy conditions. Error bars represent 1 SEM.

To investigate this possibility, we conducted a 2 (WM Demand: Low Load, High Load – Hard) x 2 (Experiment: 1, 2) mixed-design ANOVA on M_ratio_, where we use the factor name “WM Demand” rather than “WM Load” to highlight the fact that this factor now subdivides the High Load condition according to alphabetization difficulty. Indeed, we found that, compared with Low Load, High Load – Hard trials impaired M_ratio_ in both experiments [WM Demand, F(1, 49) = 9.47, *P* = 0.003; WM Demand x Experiment, *P* = 0.9; Expt 1: Low Load mean = 0.74, SEM = 0.10; High Load – Hard mean = 0.54, SEM = 0.10; Expt 2: Low Load mean = 0.68, SEM = 0.09; High Load – Hard mean = 0.46, SEM = 0.09]. By stark contrast, High Load – Easy strings did not impair M_ratio_ relative to Low Load (WM Demand, *P* > 0.9; WM Demand x Experiment, *P* > 0.7; Expt 1: Low Load mean = 0.74, SEM = 0.10; High Load – Easy mean = 0.71, SEM = 0.11; Expt 2: Low Load mean = 0.68, SEM = 0.09; High Load – Easy mean = 0.71, SEM = 0.09). M_ratio_ for High Load – Hard trials was also significantly lower than for High Load – Easy trials [WM Demand, F(1, 49) = 8.12, *P* = 0.006; WM Demand x Experiment, *P* > 0.6]. Thus, relative metacognitive sensitivity in the perceptual task was not affected by the overall memorization load placed upon WM, but rather was selectively impaired by the need to perform extensive alphabetization on High Load WM strings. These findings are portrayed in Fig. 5.

We pursued these findings further by investigating the separate effects of alphabetization difficulty under High Load on *d’* and meta-*d’*. A 2 (WM Demand: High Load – Easy, High Load – Hard) x 2 (Experiment: 1, 2) mixed-design ANOVA on *d’* did not reveal any significant effects (WM Demand, *P* = 0.12; WM Demand x Experiment, *P* = 0.19; Expt 1: High Load – Easy mean = 1.61, SEM = 0.13; High Load – Hard mean = 1.63, SEM = 0.15; Expt 2: High Load – Easy mean = 1.59, SEM = 0.11; High Load – Hard mean = 1.84, SEM = 0.13). Although the WM Demand x Experiment interaction was not significant (*P* = 0.19), one might get the visual impression from Fig. 3 that *d’* differed as a function of WM Demand in Experiment 2 but not Experiment 1. Post-hoc paired *t*-tests conducted separately for both experimental groups do not yield a significant difference in *d’* in Experiment 1 [*t*(21) = −0.2, Bonferroni corrected *P* = 1] or Experiment 2 [*t*(28) =  −1.96, Bonferroni corrected *P* = 0.12]. The marginally significant difference in *d’* for Experiment 2 goes in the opposite direction one might have expected, with *d’* being numerically higher under the High Load – Hard condition. This nonsignificant difference in *d’* in Experiment 2 does not substantively affect the primary effect of interest in this study, which is the influence of WM demand on metacognitive performance while controlling for variation in *d’*. Notably, in Experiment 2, meta-*d’* is lower under the High Load – Hard condition than in the High Load – Easy condition even though *d’* is influenced in the opposite direction.

A similar 2 (WM Demand: High Load – Easy, High Load – Hard) x 2 (Experiment: 1, 2) mixed-design ANOVA for meta-*d’* did reveal an effect of alphabetization difficulty [WM Demand, F(1, 49) = 9.31, *P* = 0.004; WM Demand x Experiment, *P* > 0.6; Expt 1: High Load – Easy mean = 1.05, SEM = 0.15; High Load – Hard mean = 0.78, SEM = 0.15; Expt 2: High Load – Easy mean = 1.07, SEM = 0.13; High Load – Hard mean = 0.71, SEM = 0.13].

Thus, whereas overall WM load impaired both *d’* and meta-*d’*, the added component of alphabetization difficulty within the High Load condition did not impair *d’*, but did impose a selective deficit for meta-*d’*.

### Confidence as a function of accuracy and WM demand

**Figure 6:**
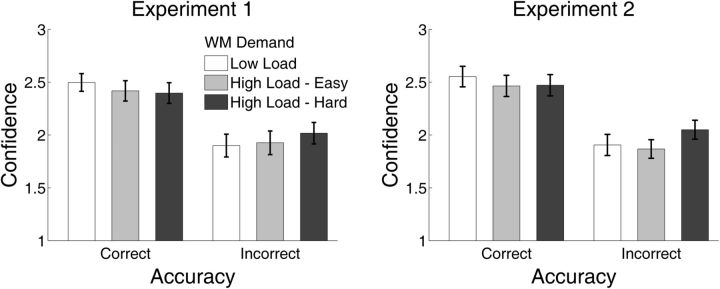
Mean levels of confidence as a function of accuracy in the perceptual task and WM load Overall levels of confidence did not differ for Low Load and High Load – Hard conditions. However, a qualitatively similar pattern arose in Experiments 1 and 2, whereby under the High Load – Hard condition, confidence for correct decisions decreased and confidence for incorrect decisions increased, relative to Low Load. Compared to the High Load – Easy condition, confidence for incorrect responses was numerically higher under the High Load – Hard condition. These patterns give a qualitative sense of the source of the differences in metacognition across the experimental conditions. Error bars represent 1 SEM.

**Figure 7:**
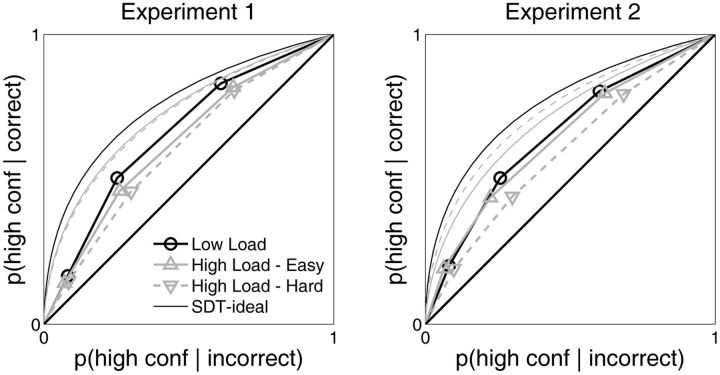
Pooled type 2 ROC curves In the analyses depicted in the previous figures, *d’* and meta-*d’* were computed separately for each subject. We supplemented this analysis by pooling together (averaging) type 2HRs [p(high conf | correct)] and type 2 false alarm rates [p(high conf | incorrect)] across subjects and using the averaged data to construct the pooled type 2 ROC curves displayed here. Thick lines depict pooled type 2 ROC data as explained in the figure legend. Thinner lines depict the SDT-ideal type 2 ROC curve for the corresponding Low Load, High Load – Easy, and High Load – Hard conditions as derived from pooled *d’*. Similar features from the main, nonpooled analyses are evident in the pooled analysis—the empirical type 2 ROC curves are closer to the diagonal line of chance metacognitive performance than are the SDT-ideal dashed curves (echoing the finding that M_ratio_ < 1), and the type 2 ROC curve is closer to chance in the High Load – Hard condition than it is under the Low Load and High Load – Easy conditions (echoing the finding that M_ratio_ is lower for the High Load – Hard condition than for Low Load and High Load – Easy). A bootstrap analysis provided quantitative statistical support for these qualitative observations (see Results).

Metacognitive sensitivity is determined by how well an observer places confidence ratings to distinguish between correct and incorrect responses. There are several ways in which High Load – Hard trials may have impaired metacognitive performance—e.g. by reducing confidence for correct responses, increasing confidence for incorrect responses, or both. To investigate, we performed a 2 (Accuracy: Correct, Incorrect) x 2 (WM Demand: Low Load, High Load – Hard) x 2 (Experiment: 1, 2) mixed-design ANOVA on confidence in the perceptual task. A significant main effect of Accuracy on confidence [F(1, 49) = 112.51, *P* < 0.001] reflects the fact that correct visual discrimination responses were associated with higher confidence. Additionally, there was a significant Accuracy x WM Demand interaction [F(1, 49) = 8.26, *P* = 0.006) which was not modulated by Experiment (Accuracy x WM Demand x Experiment, *P* > 0.6]. The Accuracy x WM Demand interaction reflects the fact that for High Load – Hard trials, confidence for correct responses decreased whereas confidence for incorrect responses increased, relative to Low Load trials (Fig. 6). Qualitatively similar patterns in confidence hold for the comparison of the High Load – Easy and High Load – Hard conditions, although in this case the Accuracy x WM Demand interaction was only marginally significant [F(1,49) = 2.75, *P* = 0.1]. This pattern can also be seen in the pooled type 2 ROC curves described in more detail in the following section (Fig. 7), as for High Load – Hard trials, type 2 false alarm rates increased whereas type 2 HRs decreased relative to Low Load and High Load – Easy trials.

We note that this analysis of the confidence data is intended to shed light on the previous results only in a qualitative way. Mean confidence data has only an indirect relationship to the type 2 ROC curves on which meta-d’ computations are based, in the sense that different type 2 ROC curves can yield the same levels of mean confidence for correct and incorrect responses. Additionally, unlike M_ratio_, mean confidence data is not adjusted for perceptual task performance. Thus, this analysis can only provide qualitative insight into the source of changes in meta-*d’* due to WM Demand.

### Pooled type 2 ROC curve analysis

One potential concern with the foregoing analyses is that trial counts were somewhat low, a concession necessary in the task design due to the relatively long duration of each trial. In Experiment 1, 200 trials contributed to the Low Load condition, and roughly 100 trials contributed to each of the High Load – Easy and High Load – Hard conditions. In Experiment 2, for each level of grating contrast, 100 trials contributed to the Low Load condition, and roughly 50 trials contributed to each of the High Load – Easy and High Load – Hard conditions. A recent investigation of the statistical properties of meta-*d’* suggests that it has acceptably low levels of bias and variance even when estimated with as few as 50 trials (Barrett *et al.*, 2013). Nonetheless, in order to lend further support to the findings described above, we performed a complementary analysis that pooled data across subjects.

The ideal approach to performing an SDT analysis is to calculate metrics such as *d’* separately for each subject, using their individual HR and false alarm rate data. But in cases where within-subject trial counts are a concern but there is ample between-subject data, an alternative approach is to average HRs and false alarm rates across subjects, and use this ‘pooled’ data to perform SDT analysis on the group as a whole (Macmillan and Kaplan, 1985; Macmillan and Creelman, 2005). This pooling approach is a legitimate way to analyze the data; for instance, it was used extensively in a classic article demonstrating SDT’s ability to characterize a wide variety of empirical ROC curves (Swets, 1986). Although the pooling approach potentially underestimates sensitivity if subjects have very different values for sensitivity or response bias (Macmillan and Kaplan, 1985), such concerns are mitigated for the present purposes, as we are primarily concerned in analyzing the ‘difference’ in metacognitive sensitivity between two conditions, rather than the overall level of metacognitive sensitivity in a single condition.

For the present purposes, we wish to compare metacognitive performance in the Low Load and the High Load – Hard conditions. Thus, we pooled data across subjects to construct pooled type 2 ROC curves. The type 2 ROC curve is a plot of type 2 HR (i.e. probability of high confidence for correct responses) against type 2 false alarm rate (i.e. probability of high confidence for incorrect responses) (Galvin *et al.*, 2003). The “type 2” designation indicates the task of classifying response accuracy with confidence ratings, in contradistinction to the “type 1” task of performing an objective classification of the stimuli. Because subjects rated confidence on a scale of 1 through 4, three (type 2 FAR, type 2 HR) pairs could be calculated for each subject by separately considering “high confidence” to consist in all confidence ratings >1, all ratings >2 or all ratings >3 (Macmillan and Creelman, 2005). We computed the (type 2 FAR, type 2 HR) pairs for each subject in the Low Load and High Load – Hard conditions and averaged these across subjects. The resulting ROC curves are displayed in Fig. 7. We similarly computed the across-subject average (FAR, HR) for the visual discrimination task, and computed a group *d’* from this pooled data. We used this value of pooled *d’* to construct the ideal pooled type 2 ROC curve, assuming unbiased responding in the visual discrimination task (Maniscalco and Lau, 2012).

Visual inspection of the pooled type 2 ROC curves confirms that metacognitive performance was worse under the High Load – Hard condition than under the Low Load and High Load – Easy conditions, as under this condition the type 2 ROC curve lies closer to the line of chance metacognitive performance, i.e. the line where type 2 FAR = type 2 HR.

In order to quantify this observation, we performed a bootstrap analysis (Mooney and Duval, 1993). In the bootstrap procedure, the sampling distribution for a variable is estimated by repeatedly resampling with replacement from the original data set and computing the value of the variable for each such bootstrap sample. We constructed 1000 bootstrap samples of the type 1 and type 2 HR and false alarm rate data for each WM Demand condition. For each bootstrap sample, we calculated *d’* and estimated meta-*d’* by finding the least-squares fit of the meta-*d’* model to the type 2 ROC curve (Maniscalco and Lau, 2012). We then analyzed the distribution of values for M_D, L_ =  M_Low Load_ – M_High Load – Hard_ and M_D, H_ = M_High Load – Easy_ – M_High Load – Hard_. For Experiment 1, the mean M_D, L_ was 0.21 and only 3.9% of all bootstrap samples had M_D, L_ < 0, and for Experiment 2, the mean M_D, L_ was 0.28 and only 2% of all bootstrap samples had M_D, L_ < 0. Similarly, for Experiment 1, the mean M_D, H_ was 0.16 and only 6% of all bootstrap samples had M_D, H_ < 0, and for Experiment 2, the mean M_D, H_ was 0.30 and only 1.6% of all bootstrap samples had M_D, H_ < 0. Thus, this complementary bootstrap analysis of the pooled type 2 ROC data provides converging evidence for the claim that metacognitive performance was impaired under the High Load – Hard condition relative to the Low Load and High Load – Easy conditions.

## Discussion

In summary, we found that when human subjects performed a WM task concurrently with a perceptual decision making task, performance on the two tasks interacted in interesting ways. First, there was an overall effect of WM load whereby both perceptual (*d’*) and metacognitive (meta-*d’*) sensitivity in the perceptual task decreased when longer letter strings had to be maintained in memory. Second, there was a specific effect of the manipulation demand imposed by WM contents on perceptual metacognition. For letter strings that were initially poorly alphabetized, stronger manipulation demand was imposed upon subjects, as they had to perform more mental operations upon WM contents in order to arrive at a properly alphabetized string. This manipulation demand had selective effects upon relative metacognitive sensitivity, as measured by M_ratio_ = meta-*d’*/*d’*. When manipulation demand for four-letter WM strings was low (High Load – Easy trials), M_ratio_ did not differ for high and low WM load. But when manipulation demand for four-letter strings was high (High Load – Hard trials), M_ratio_ was significantly lower than in the Low Load and High Load – Easy conditions. Thus, relative metacognitive sensitivity was insensitive to overall WM load, but was selectively impaired when extensive manipulation of WM contents was required.

It is important to interpret these results in light of the theoretical distinction between ‘absolute’ and ‘relative’ metacognitive sensitivity introduced in Maniscalco and Lau (2012). Absolute metacognitive sensitivity refers to the overall efficacy with which confidence ratings discriminate between correct and incorrect responses, as measured e.g. by area under the type 2 ROC curve (Galvin *et al.*, 2003; Fleming *et al.*, 2010). Relative metacognitive sensitivity, or metacognitive efficiency (Fleming and Lau, 2014), evaluates the empirically observed level of absolute metacognitive sensitivity with respect to the ‘expected’ level of absolute metacognitive sensitivity, given an observer’s performance on the primary stimulus classification task. Such an expectation can be derived by the theoretical machinery of SDT (Galvin *et al.*, 2003), with the important features that (i) task performance should place a theoretical limit on metacognitive performance [but see Scott *et al.* (2014)], and (ii) as task performance improves, so should metacognitive performance. These theoretical predictions have been validated in empirical data (Maniscalco and Lau, 2012).

The SDT measure of absolute metacognitive sensitivity, meta-*d’* (Maniscalco and Lau, 2012, 2014), was designed with an eye toward providing a straightforward way to measure relative metacognitive sensitivity. Meta-*d’* is defined such that, for an observer whose performance conforms to SDT assumptions, meta-*d’* = *d’*. Thus, relative metacognitive sensitivity can be operationalized as a direct numerical comparison between meta-*d’* and *d’*, e.g. a subtraction or division. In this study, we found evidence that the y-intercept of the function relating meta-*d’* and *d’* is zero (Fig. 3). On the assumption that the relationship between *d’* and meta-*d’* is linear with a y-intercept of zero, the slope of the line provides a constant measure of metacognitive sensitivity across different levels of *d’* and can be calculated as the simple ratio meta-*d’* / *d’*. Thus, using meta-*d’* / *d’* as the measure of relative metacognitive sensitivity for these data is appropriate, given its consistency with the empirical data. For instance, if we suppose that the true function relating meta-d’ and d’ for a constant level of metacognitive sensitivity is given by meta-*d’* = 0.8 * *d’*, then the ratio meta-*d’* / *d’* would have a constant value of 0.8 regardless of the value of *d’* and reflect the true underlying metacognitive sensitivity, whereas the value of the difference meta-*d’* – *d’* would erroneously differ depending on the value of *d’*.

In the current data set, although meta-*d’* decreased under high WM load, *d’* also decreased to a similar extent. Given the known theoretical and empirical dependence of meta-*d’* upon *d’* (Galvin *et al.*, 2003; Maniscalco and Lau, 2012), it is therefore possible to attribute the decline in meta-*d’* under high WM load to the co-occurring decline in *d’*, rather than supposing that WM load had a direct effect upon metacognition. Indeed, the relative measure of metacognitive sensitivity, meta-*d’*/*d’*, did not significantly differ as a function of WM load. Thus, while high WM load imposed an overall deficit in performance on the perceptual task, perhaps due to reduced attentional allocation to the visual stimuli under High Load, we did not find strong evidence that WM load produced a selective deficit upon metacognitive processing in and of itself.

In contrast, we found that relative metacognitive sensitivity in the perceptual task was indeed impaired in the specific case where the contents of WM required a substantial degree of manipulation (alphabetization). This finding is in keeping with prior empirical investigations on the neural bases of visual metacognition and WM performance. Various higher-level regions of the human and monkey PFC, includingdlPFC, rlPFC and aPFC, have been linked to metacognitive performance in visual and memory tasks (Henson *et al.*, 2000; Fleck *et al.*, 2006; Fleming *et al.*, 2010; Tsujimoto *et al.*, 2010; Yokoyama *et al.*, 2010; Fleming *et al.*, 2012; McCurdy *et al.*, 2013). Similarly, dlPFC in particular has been linked to performance in WM tasks, with a strong line of evidence that dlPFC is involved particularly with the ‘manipulation’ and ‘selection’ of WM contents, rather than just the passive maintenance of items in WM (Petrides and Milner, 1982; Petrides, 1989, 1995; Owen *et al.*, 1996; D'Esposito *et al.*, 1999; Petrides, 2000; Rowe *et al.*, 2000; Miller and Cohen, 2001; Rowe and Passingham, 2001; Rowe *et al.*, 2002; Bor *et al.*, 2003; Curtis and D'Esposito, 2003). In the current study, the fact that relative metacognitive sensitivity was impaired not by overall WM load, but rather by the specific requirement to extensively manipulate WM contents, suggests that a common cognitive mechanism may contribute to both the manipulation of WM contents and the metacognitive evaluation of visual task performance. In turn, the neuroscience literature suggests that such a common mechanism may be instantiated by the dlPFC.

We note that a somewhat similar finding to the current study was previously reported in the context of visual search tasks by Han and Kim (2004). In that study, the time required to find a visual target in a cluttered display as a function of display set size (search slope) was compared for concurrent WM tasks that either did or did not require active manipulation of WM contents (backwards counting for number items, or alphabetization for letter items). Search slope was significantly steeper than in a control condition when subjects had to manipulate WM contents, but not when subjects had to passively maintain a number or letter string in WM. Thus, as in the present study, Han and Kim found that aspects of processing in a visual task could undergo selective impairment due to the requirement to manipulate WM contents. Han and Kim concluded that aspects of executive functioning, as reflected in manipulation of WM contents, may be required to perform visual search. However, it is unclear to what extent this impairment in visual search is related to the impairment on relative metacognitive sensitivity observed in the current study.

Future work should seek to generalize and extend the present findings. Following previous studies (D'Esposito *et al.*, 1999; Han and Kim, 2004), our task design required subjects to alphabetize letter strings held in WM in order to study the more general cognitive operation of online manipulation of WM contents. We speculate that any task requiring online manipulation of WM contents would exhibit similar interference with metacognition in a concurrent visual task, but this remains to be demonstrated. Additionally, in the present work we have explored only a binary contrast in difficulty of the WM manipulation task (High Load – Easy vs High Load – Hard conditions). These results could be extended by a more parametric modulation of the difficulty of manipulating WM contents. A simple extrapolation from the current findings would predict that continuous increases in WM manipulation difficulty would be accompanied by continuous decreases in relative metacognitive sensitivity.

How might it be the case that a cognitive/neural mechanism that contributes to manipulation of WM contents also contributes to metacognitive evaluation of visual perception? The explanation cannot be an overly general mechanism, such as supposing that additional attentional resources required for the WM task would leave fewer attentional resources for the visual task. Such general mechanisms would presumably induce global changes in visual task performance, affecting both *d’* and meta-*d’*, rather than being specific to meta-*d’* / *d’*.

One potentially trivializing account is that the task design artificially required subjects to use WM mechanisms to evaluate confidence, since subjects indicated their perceptual decision about the visual stimulus with an initial key press and then indicated decision confidence with a second key press. An alternative task design, e.g. one that allowed subjects to indicate perceptual decision and decision confidence simultaneously, might impose less WM demand on the confidence rating. However, this concern is unlikely to account for the present findings. Subjects typically indicated confidence following the initial perceptual decision in fewer than 400 ms (average of median RT for each subject = 387 ms in Experiment 1 and 381 ms in Experiment 2), and median confidence RT following perceptual decision was not affected by WM load (paired *t*-tests on median confidence RTs for Experiments 1 and 2, *p*s > 0.7). Thus, the temporal duration of any extra WM demand potentially imposed by the second key press was minimal and unaffected by WM load imposed by the primary memory task. The fast confidence RTs also suggest that subjects may have largely formed their confidence ratings even prior to the first key press about the stimulus itself, rather than delaying the formation of the confidence rating until after the first key press. Importantly, any extra WM load attributable to the process of forming a confidence rating cannot explain the primary finding of the current results, which is that active manipulation of WM contents, rather than passive storage, is what selectively interferes with metacognitive sensitivity. In particular, in comparisons of the High Load – Easy and High Load – Hard conditions, WM load incurred by both the letter string and the perceptual task is equivalent, so differences in metacognition in this contrast can only be attributed to the requirement to actively manipulate WM contents.

One potential mechanism might have to do with strategies for representing and re-representing stimuli. In WM tasks, lateral PFC activation has been associated with encoding strategies for WM contents—when items are presented in a way that facilitates their reorganization into higher-level units or “chunks,” lateral PFC becomes more activated (Bor *et al.*, 2003). Some views hold that metacognition similarly involves the construction of higher-order re-representations or meta-representations of cognitive/neural processing occurring at lower levels in the processing hierarchy (Nelson and Narens, 1990; Schooler, 2002; Cleeremans *et al.*, 2007; Pasquali *et al.*, 2010). If so, it is possible that the same processes involved in manipulating and reorganizing WM contents might also be involved in manipulating and reorganizing sensory representations for the purposes of metacognitive evaluation. Presumably, occupation of such a resource in the manipulation of WM contents would detract from its active employment in the metacognitive evaluation of visual processing, thus impairing relative metacognitive sensitivity.

Another possible set of common underlying mechanisms concerns response selection and the maintenance and flexible adaptation of decision rules. Response selection, defined by Curtis and D’Esposito (2003) as “the operation by which information in short-term storage becomes the focus of attention such that it can be maintained and eventually used to choose an appropriate motor response” (p. 421), has been tied to dlPFC activity in the context of WM tasks (Rowe *et al.*, 2000; Rowe and Passingham, 2001; Rowe *et al.*, 2002; Curtis and D'Esposito, 2003). More broadly, PFC has been theorized to support varying levels of sophistication and abstraction in the control and organization of behavior as a function of stimuli and environmental context, action contingencies, currently active goals and so on (Koechlin and Summerfield, 2007; Badre, 2008). By way of comparison, the SDT model posits that perceptual classification of stimuli and confidence ratings are the outcomes of cognitive decision processes that are not the rigid outcome of low-level perceptual processing but rather can be flexibly adjusted according to the prevailing task instructions, stimulus context and reward contingencies (Tanner and Swets, 1954; Green and Swets, 1966; Macmillan and Creelman, 2005). According to SDT, perceptual and metacognitive decisions are determined by defining a set of decision criteria which determine the rules according to which graded and ambiguous internal perceptual evidence is mapped onto discrete perceptual decisions and motor outputs (Green and Swets, 1966; Macmillan and Creelman, 2005). A common mechanism in PFC underlying the processes of selecting, evaluating and manipulating WM contents in the WM task and the processes of metacognitive criterion setting in the perceptual task could potentially explain the results of the current study. Specifically, response selection mechanisms may be more taxed in experimental conditions where the letter string in WM requires more extensive alphabetization; if similar response selection mechanisms govern the process of metacognitive evaluation and criterion setting, then the burden of more extensive alphabetization in the WM task would result in poorer metacognitive efficiency in the perceptual task. The common cognitive theme for both evaluating WM contents by a process of mental manipulation and evaluating decision confidence via criterion setting is that in both processes, the underlying mental content (letter string in WM, or strength of perceptual confidence) is not sufficient to determine a behavioral response, but rather the behavioral response must be mediated by an evaluative decision-making process.

Given that perceptual decision making also requires application of decision rules to sensory evidence, one might wonder what makes the above criterion setting account specific to ‘metacognitive’ criterion setting. One possibility is that perceptual criterion setting for the 2AFC task used in the current study is relatively trivial compared to the metacognitive criterion setting, and therefore is robust to interference from the WM task. If so, it is possible that more challenging perceptual decision making task structures (e.g. visual detection tasks) might exhibit performance deficits under conditions requiring manipulation of WM content. In a detection task, as in confidence rating, to determine an optimal criterion one needs to have some knowledge of the statistics regarding the overall signal strength and noise level. In contrast, in a 2AFC task, the optimal strategy is simply to base one’s response on the sign of the difference of the sensory evidence for the two alternatives, and this strategy does not require knowledge of the statistical properties of sensory evidence across trials.

Alternatively, the distinction between perceptual and metacognitive decision making might be grounded in neuroanatomy. Perceptual decision making has been associated with more posterior regions of PFC (Heekeren *et al.*, 2006, 2008), whereas visual metacognition is subserved by more anterior regions of PFC (Fleming *et al.*, 2010; Rounis *et al.*, 2010; Fleming *et al.*, 2012, 2014). Since manipulating and applying decision rules to WM contents engages more anterior parts of PFC (D'Esposito *et al.*, 1999; Rowe *et al.*, 2000; Rowe and Passingham, 2001), it may therefore selectively interfere with neural resources dedicated to metacognitive decision making but not general perceptual decision making. Notably, Heekeren *et al.* (2006) found perceptual decision making to be associated with the posterior DLPFC in Broadmann Area (BA) 8/9, whereas Rowe *et al.* (2000) found that BA 8 was associated with passive WM maintenance and it was the more anterior BA 46 that was associated with response selection on the basis of WM contents.

Regardless of the specific manner in which manipulation of WM contents influences metacognitive performance, the results of this study demonstrate a dissociation between perceptual and metacognitive sensitivity, suggesting that these depend on separate underlying mechanisms. Indirect evidence for such a position comes from anatomical (Fleming *et al.*, 2010; McCurdy *et al.*, 2013) and fMRI (Henson *et al.*, 2000; Fleck *et al.*, 2006; Yokoyama *et al.*, 2010; Fleming *et al.*, 2012) studies in humans, and single-unit recordings in monkeys (Tsujimoto *et al.*, 2010), which associate metacognitive performance with high-level structures in PFC rather than earlier visual processing regions. Direct evidence comes from studies demonstrating that when PFC function is impaired by transcranial magnetic stimulation or lesion, metacognitive sensitivity in visual tasks is selectively disrupted (Rounis *et al.*, 2010; Fleming *et al.*, 2014). Here we provide another line of direct evidence for a perceptual/metacognitive dissociation by demonstrating the existence of a purely cognitive, task-based intervention that selectively disrupts visual metacognitive performance. 

## References

[niv002-B1] Badre D Cognitive control, hierarchy, and the rostro-caudal organization of the frontal lobes. Trends Cogn Sci 2008;12:193–200. 18403252 10.1016/j.tics.2008.02.004

[niv002-B2] Barrett AB Dienes Z Seth AK Measures of metacognition on signal-detection theoretic models. Psychol Methods 2013;18:535–52. 24079931 10.1037/a0033268

[niv002-B3] Bor D Duncan J Wiseman RJ Encoding strategies dissociate prefrontal activity from working memory demand. Neuron 2003;37:361–67. 12546829 10.1016/s0896-6273(02)01171-6

[niv002-B4] Brainard DH The psychophysics toolbox. Spat Vis 1997;10:433–36. 9176952

[niv002-B5] Curtis CE D'Esposito M Persistent activity in the prefrontal cortex during working memory. Trends Cogn Sci 2003;7:415–23. 12963473 10.1016/s1364-6613(03)00197-9

[niv002-B6] Cleeremans A Timmermans B Pasquali A Consciousness and metarepresentation: a computational sketch. Neural Netw 2007;20:1032–39. 17904799 10.1016/j.neunet.2007.09.011

[niv002-B7] D'Esposito M Postle BR Ballard D Maintenance versus manipulation of information held in working memory: an event-related fMRI study. Brain Cogn 1999;41**:** 66–86. 10536086 10.1006/brcg.1999.1096

[niv002-B8] Fleck MS Daselaar SM Dobbins IG Role of prefrontal and anterior cingulate regions in decision-making processes shared by memory and nonmemory tasks. Cereb Cortex 2006;16:1623–30. 16400154 10.1093/cercor/bhj097

[niv002-B9] Fleming SM Huijgen J Dolan RJ Prefrontal contributions to metacognition in perceptual decision making. J Neurosci 2012;32, 6117–25. 22553018 10.1523/JNEUROSCI.6489-11.2012 PMC3359781

[niv002-B10] Fleming SM Lau H How to measure metacognition. Front Hum Neurosci 2014;8, 443. 25076880 10.3389/fnhum.2014.00443 PMC4097944

[niv002-B11] Fleming SM Ryu J Golfinos JG Domain-specific impairment in metacognitive accuracy following anterior prefrontal lesions. Brain 2014;137:2811–22. 25100039 10.1093/brain/awu221 PMC4163038

[niv002-B12] Fleming SM Weil RS Nagy Z Relating introspective accuracy to individual differences in brain structure. Science 2010;329:1541–43. 20847276 10.1126/science.1191883 PMC3173849

[niv002-B13] Galvin SJ Podd JV Drga V Type 2 tasks in the theory of signal detectability: discrimination between correct and incorrect decisions. Psychon Bull Rev 2003;10:843–76. 15000533 10.3758/bf03196546

[niv002-B14] Green DM Swets JA Signal Detection Theory and Psychophysics. New York: Wiley, 1966.

[niv002-B15] Han SH Kim MS Visual search does not remain efficient when executive working memory is working. Psychol Sci 2004;15:623–28. 15327634 10.1111/j.0956-7976.2004.00730.x

[niv002-B16] Heekeren HR Marrett S Ruff DA Involvement of human left dorsolateral prefrontal cortex in perceptual decision making is independent of response modality. Proc Natl Acad Sci USA 2006;103:10023–28. 16785427 10.1073/pnas.0603949103 PMC1479865

[niv002-B17] Heekeren HR Marrett S Ungerleider LG The neural systems that mediate human perceptual decision making. Nat Rev Neurosci 2008;9:467–79. 18464792 10.1038/nrn2374

[niv002-B18] Henson RN Rugg MD Shallice T Confidence in recognition memory for words: dissociating right prefrontal roles in episodic retrieval. J Cogn Neurosci 2000;12:913–23. 11177413 10.1162/08989290051137468

[niv002-B19] Koechlin E Summerfield C An information theoretical approach to prefrontal executive function. Trends Cogn Sci 2007;11:229–35. 17475536 10.1016/j.tics.2007.04.005

[niv002-B20] Macmillan NA Creelman CD Detection Theory: A User’s Guide*,* 2nd edn United States of America: Lawrence Erlbaum, 2005.

[niv002-B21] Macmillan NA Kaplan HL Detection theory analysis of group data: estimating sensitivity from average hit and false-alarm rates. Psychol Bull 1985;98:185–99. 4034817

[niv002-B22] Maniscalco B Lau H A signal detection theoretic approach for estimating metacognitive sensitivity from confidence ratings. Conscious Cogn 2012;21:422–30. 22071269 10.1016/j.concog.2011.09.021

[niv002-B23] Maniscalco B Lau H Signal detection theory analysis of type 1 and type 2 data: meta-d’, response-specific meta-d’, and the unequal variance SDT model. In: Fleming SM Frith CD (eds.), The Cognitive Neuroscience of Metacognition. Heidelberg New York Dordrecht London: Springer, 2014, 25–66.

[niv002-B24] Marois R Ivanoff J Capacity limits of information processing in the brain. Trends Cogn Sci 2005;9:296–305. 15925809 10.1016/j.tics.2005.04.010

[niv002-B25] McCurdy LY Maniscalco B Metcalfe J Anatomical coupling between distinct metacognitive systems for memory and visual perception. J Neurosci 2013; 33:1897–906. 23365229 10.1523/JNEUROSCI.1890-12.2013 PMC4696871

[niv002-B26] Miller EK Cohen JD An integrative theory of prefrontal cortex function. Annu Rev Neurosci 2001;24:167–202. 11283309 10.1146/annurev.neuro.24.1.167

[niv002-B27] Mooney CZ Duval RD Bootstrapping: A Nonparametric Approach to Statistical Inference. Newbury Park, CA: Sage Publications, 1993.

[niv002-B28] Nelson TO Narens L Metamemory: a theoretical framework and new findings. Psychol Learn Motivat Adv Res Theory 1990;26:125–73.

[niv002-B29] Owen AM Morris RG Sahakian BJ Double dissociations of memory and executive functions in working memory tasks following frontal lobe excisions, temporal lobe excisions or amygdalo-hippocampectomy in man. Brain 1996;119:1597–615. 8931583 10.1093/brain/119.5.1597

[niv002-B30] Pasquali A Timmermans B Cleeremans A Know thyself: metacognitive networks and measures of consciousness. Cognition 2010;117:182–90. 20825936 10.1016/j.cognition.2010.08.010

[niv002-B31] Pelli DG The VideoToolbox software for visual psychophysics: transforming numbers into movies. Spat Vis 1997;10:437–42. 9176953

[niv002-B32] Petrides M Frontal lobes and memory. In: Boller F Grafman J (eds.), Handbook of Neuropsychology, vol. 3 Amsterdam: Elsevier, 1989, 75–90.

[niv002-B33] Petrides M Impairments on nonspatial self-ordered and externally ordered working memory tasks after lesions of the mid-dorsal part of the lateral frontal cortex in the monkey. J Neurosci 1995;15: 359–75. 7823141 10.1523/JNEUROSCI.15-01-00359.1995 PMC6578311

[niv002-B34] Petrides M The role of the mid-dorsolateral prefrontal cortex in working memory. Exp Brain Res 2000;133:44–54. 10933209 10.1007/s002210000399

[niv002-B35] Petrides M Milner B Deficits on subject-ordered tasks after frontal- and temporal-lobe lesions in man. Neuropsychologia 1982;20:249–62. 7121793 10.1016/0028-3932(82)90100-2

[niv002-B36] Rounis E Maniscalco B Rothwell JC Theta-burst transcranial magnetic stimulation to the prefrontal cortex impairs metacognitive visual awareness. Cogn Neurosci 2010;1:165–75. 24168333 10.1080/17588921003632529

[niv002-B37] Rowe JB Friston K Frackowiak R Attention to action: specific modulation of corticocortical interactions in humans. Neuroimage 2002;17:988–98. 12377172

[niv002-B38] Rowe JB Passingham RE Working memory for location and time: activity in prefrontal area 46 relates to selection rather than maintenance in memory. Neuroimage 2001;14:77–86. 11525340 10.1006/nimg.2001.0784

[niv002-B39] Rowe JB Toni I Josephs O The prefrontal cortex: response selection or maintenance within working memory? Science 2000;288:1656–60. 10834847 10.1126/science.288.5471.1656

[niv002-B40] Schooler JW Re-representing consciousness: dissociations between experience and meta-consciousness. Trends Cogn Sci 2002;6:339–44. 12140084 10.1016/s1364-6613(02)01949-6

[niv002-B41] Scott RB Dienes Z Barrett AB Blind insight: metacognitive discrimination despite chance task performance. Psychol Sci 2014;25:2199–208. 25384551 10.1177/0956797614553944 PMC4263819

[niv002-B42] Swets JA Form of empirical ROCs in discrimination and diagnostic tasks: implications for theory and measurement of performance. Psychol Bull 1986;99:181–98. 3515382

[niv002-B43] Tanner WP Jr Swets JA A decision-making theory of visual detection. Psychol Rev 1954; 61:401–9. 13215690 10.1037/h0058700

[niv002-B44] Tsujimoto S Genovesio A Wise SP Evaluating self-generated decisions in frontal pole cortex of monkeys. Nat Neurosci 2010;13:120–26. 19966838 10.1038/nn.2453 PMC2834888

[niv002-B45] Watson AB Pelli DG QUEST: a Bayesian adaptive psychometric method. Percept Psychophys 1983;33:113–20. 6844102 10.3758/bf03202828

[niv002-B46] Yokoyama O Miura N Watanabe J Right frontopolar cortex activity correlates with reliability of retrospective rating of confidence in short-term recognition memory performance. Neurosci Res 2010;68:199–206. 20688112 10.1016/j.neures.2010.07.2041

